# Defoliating Insect Mass Outbreak Affects Soil N Fluxes and Tree N Nutrition in Scots Pine Forests

**DOI:** 10.3389/fpls.2017.00954

**Published:** 2017-06-07

**Authors:** Maren M. Grüning, Judy Simon, Heinz Rennenberg, Anne l-M-Arnold

**Affiliations:** ^1^Department of Soil Science of Temperate Ecosystems, Georg-August Universität GöttingenGöttingen, Germany; ^2^Ecology, Department of Biology, University of KonstanzKonstanz, Germany; ^3^Chair of Tree Physiology, Institute of Forest Sciences, University of FreiburgFreiburg, Germany; ^4^King Saud UniversityRiyadh, Saudi Arabia

**Keywords:** feces, inorganic N, litter, N fluxes, nitrate, N metabolites, organic N, throughfall

## Abstract

Biotic stress by mass outbreaks of defoliating pest insects does not only affect tree performance by reducing its photosynthetic capacity, but also changes N cycling in the soil of forest ecosystems. However, how insect induced defoliation affects soil N fluxes and, in turn, tree N nutrition is not well-studied. In the present study, we quantified N input and output fluxes via dry matter input, throughfall, and soil leachates. Furthermore, we investigated the effects of mass insect herbivory on tree N acquisition (i.e., organic and inorganic ^15^N net uptake capacity of fine roots) as well as N pools in fine roots and needles in a Scots pine (*Pinus sylvestris* L.) forest over an entire vegetation period. Plots were either infested by the nun moth (*Lymantria monacha* L.) or served as controls. Our results show an increased N input by insect feces, litter, and throughfall at the infested plots compared to controls, as well as increased leaching of nitrate. However, the additional N input into the soil did not increase, but reduce inorganic and organic net N uptake capacity of Scots pine roots. N pools in the fine roots and needles of infested trees showed an accumulation of total N, amino acid-N, protein-N, and structural N in the roots and the remaining needles as a compensatory response triggered by defoliation. Thus, although soil N availability was increased via surplus N input, trees did not respond with an increased N acquisition, but rather invested resources into defense by accumulation of amino acid-N and protein-N as a survival strategy.

## Introduction

In forest ecosystems, nitrogen (N) cycling is influenced by insect herbivory (e.g., Kurz et al., 2008; Le Mellec and Michalzik, 2008; Morehouse et al., 2008; Cobb et al., 2010) already at low to moderate levels via loss of foliage, tree growth, throughfall leaching, litterfall, and litter decomposition (Schowalter et al., 1991; Chapman et al., 2003; Cunningham et al., 2009). With rapid defoliation by phytophagous insects, large amounts of tree biomass and thus nutrients are turned first into insect biomass and are subsequently released to the soil (Russel et al., 2004). To date, only few studies have analyzed the direct links between insect mass outbreaks and N fluxes from canopy to soil as well as nutrient dynamics and partitioning in the rhizosphere (e.g., Pederson and Bille-Hanssen, 1995; Le Mellec and Michalzik, 2008; Pitman et al., 2010). Consequently, only little is known about alterations of resource allocation patterns by herbivory (Kaitaniemi et al., 1999; Sampedro et al., 2009).

In forest ecosystems, insect mass outbreaks result in the mobilization of large amounts of organic N previously stored in otherwise long-living needles, which reenter ecosystem N cycling within a relatively short amount of time as a consequence of foliage loss. Thus, N entry to the soil is increased directly and indirectly via decomposition processes (Hunter, 2001; Lovett et al., 2002; Keville et al., 2013) influencing microbial community structure, GHG flux, and N turnover (Michalzik and Stadler, 2000; Stremińska et al., 2006; Le Mellec et al., 2011). This organic N input originates from both dissolved N from throughfall (Le Mellec and Michalzik, 2008) as well as solid deposition of insect feces, dead larvae, and leaf fragments (Christenson et al., 2002; Le Mellec et al., 2009; Kaňa et al., 2013). Focusing on organic N, increasing amounts are based on two processes: (1) Partly eaten needle fragments are dropped by the feeding larvae, adding additional N to the needle litter. (2) Total N concentration in the needles of infested trees is increased compared to non-infested trees as a compensatory response to frass. For example, needles of Masson pine (*Pinus massoniana* Lamb.) infested with Masson pine moth (*Dendrolimus punctatus* Walker) accumulate secondary metabolites as chemical defense measures (Fang et al., 2016). Consequently, total needle N content is increased, in turn changing the composition of future litter (Millard and Grelet, 2010). Furthermore, compared to leaf litter, insect feces often have an altered chemical as well as physical quality due to larvae digestion which causes inhomogeneous and porous surfaces and a wider C:N ratio (Le Mellec et al., 2009). In the soil, this surplus of N in combination with quality changes can accelerate important processes in N cycling, i.e., (1) soil respiration (Reynolds and Hunter, 2001; Frost and Hunter, 2004), which might lead to increased soil CO_2_ emissions (Lovett and Ruesink, 1995; Michalzik and Stadler, 2000), (2) mineralization, especially in N-limited ecosystems (Belovsky and Slade, 2000; Chapman et al., 2003; Le Mellec and Michalzik, 2008; Heinzdorf, 2013), and (3) N leaching, particularly of nitrate, causing further N losses to the system (Swank et al., 1981; Pitman et al., 2010; Le Mellec et al., 2011). In contrast to these effects accelerating N turnover, other studies suggest a redistribution of N in the soil with no detectable ammonia volatilization, nitrous oxide emission, or nitrate leaching (Russel et al., 2004) and/or soil microbial N immobilization due to a wider C:N ratio. This might be observed especially when organic material, such as feces, are supplied to the soil, indicating slowed decomposition rates due to limited N availability (Le Mellec et al., 2009; Katayama et al., 2014).

The loss of major amounts of biomass as a consequence of insect mass outbreaks not only affects ecosystem N cycling, but also mediates tree internal changes (Dale et al., 2001; Kurz et al., 2008), such as nutrition, especially with regard to N and water relations (Kosola et al., 2001; Morehouse et al., 2008). However, contradictory results on the consequences of herbivory on N nutrition and water relations of forest trees have been reported. For example, external input of N via atmospheric deposition or fertilization over several years, and increased N storage were observed in eastern hemlock (Gómez et al., 2012; Rubino et al., 2015) and red oak (Frost and Hunter, 2008). N storage was mostly a result of free amino acid accumulation (especially arginine and glutamine; Vestgarden, 2001; Throop and Lerdau, 2004; Kos et al., 2015). Increased soil N availability can lead to increased N uptake by tree roots (e.g., Stoelken et al., 2010; Li et al., 2015). However, in response to insect mass outbreaks, inorganic N uptake capacity was reduced in hybrid poplar (*Populus* × *canadensis* cv. Eugeneii) defoliated by gypsy moth (*Lymantria dispar* L.) (Kosola et al., 2001). In contrast, studies investigating eastern hemlock (*Tsuga canadensis* L.) and red oaks (*Quercus rubra* L.) found no effect of defoliation on inorganic N uptake (Lovett and Tobiessen, 1993; Rubino et al., 2015). Loss of leaf biomass also changes tree internal water relations via a reduction in leaf transpiration, thereby negatively affecting tree water balance and nutrient uptake (Aroca et al., 2012). Until today, it is unknown whether organic N uptake capacity is affected by insect mass outbreaks and if infested trees have altered preferences for inorganic or organic N sources. However, organic N sources contribute significantly to tree N nutrition, particularly in N-limited forest ecosystems (Stoelken et al., 2010; Dong et al., 2015, 2016; Li et al., 2015). Tree internal changes in physiology often result in decreasing growth rates and higher mortality (Kosola et al., 2001; Morehouse et al., 2008). However, also compensatory effects are observed including increased biomass production and higher growth rates (Russel et al., 2004) or N accumulation in different plant parts, mostly as proteins and amino acids (Gómez et al., 2012; Rubino et al., 2015), thereby increasing the nutritional value for herbivores. Particularly with regard to N, external uptake and internal allocation of N, but also a shift to C in the C/N ratio can support defense measures of trees (e.g., via the production of phenolics; Frost and Hunter, 2008), and thereby directly affect insect population levels, survivorship, as well as outbreak frequency (Throop and Lerdau, 2004).

Here, we study the effects of a mass outbreak of nun moth (*Lymantria monacha* L.)—a member of the Lymantriidae family of Lepidoptera—on N fluxes in the soil and the consequences for N nutrition of Scots pine (*Pinus sylvestris* L.) over the course of a year. In Germany, the nun moth is widely distributed in the pine forest of the north-eastern lowlands (Majunke et al., 2002). In spring 2013, 5,800 ha of forest in Brandenburg were affected by mass outbreaks of the nun moth including 366 ha of total defoliation (Möller and Heydeck, 2013). At that time, more than 11,000 ha of Scots pine forest were treated with insecticides (Möller and Heydeck, 2013). The study aimed to characterize the effects of insect mass outbreaks (1) on soil N fluxes (i.e., in litter, insect feces, throughfall, leachates), (2) on inorganic and organic N acquisition strategies of Scots pine, (3) on compensatory processes in the N nutritional status of fine roots and needles, (4) In addition, it was assessed how these responses shift over time. For this purpose, we quantified N contents in throughfall, dry matter input, soil leachates, and metabolites in fine rots and needles (i.e., total soluble amino acid, total soluble protein, and structural N levels). In addition, we quantified inorganic and organic ^15^N net uptake capacity of fine roots. We hypothesize that (1) N input via solid and wet depositions and N leaching via the soil solution will increase during the insect mass outbreak, especially during the main defoliation period (Le Mellec et al., 2009; Kaňa et al., 2013; Keville et al., 2013). (2) Infested trees are physiologically unable to take advantage of the fertilization effect of additional litter, because the uptake of nutrients and water is restricted by the loss of needle biomass, thereby leading to a decreased N uptake capacity (Kosola et al., 2001). (3) In fine roots and needles, amounts of total soluble proteins and amino acids increase as a compensatory effect to needle biomass loss (Frost and Hunter, 2008; Gómez et al., 2012; Rubino et al., 2015).

## Materials and methods

### Field site description

To study the consequences of insect mass outbreaks on soil N fluxes and tree N nutrition in Scots pine forests, two forest districts were chosen. One district was infested by the nun moth (*L. monacha* L.), the other served as control. Within each district, three plots of 300–350 m^2^ were selected. The infested district was located 3 km north of Märkisch Buchholz, Brandenburg, Germany (52°8′38″N, 13°45′14″E, 42 m a. s. l.); the control district—a stand with comparable initial site conditions but with an uncritical abundance of insect pests—10 km west of Teupitz, Brandenburg, Germany (52°9′29″N, 13°36′47″E, 35 m a. s. l.). Following the administrative procedure of forest protection for monitoring of pests (nun moth of 25.05.93) in the study area, nun moth calamity was classified “heavy” (based on 1,185 counted moths on four representative stems) in 2013 with a needle loss of ~80% according to the Eberswalde Forestry State Center of Excellence (Landeskompetenzzentrum Forst Eberswalde, unpublished data). These mass outbreaks of nun moth can be favored by the semi-arid conditions of the study area, because increased frequencies of nun moth mass outbreaks are related with drought stress (Bejer, 1988). All six plots were located in 65-year-old white moss pine forest (*Leucobryo-Pinetum*) stands with a total abundance of 96% Scots pine (*P. sylvestris* L.) and 4% beech (*Fagus sylvatica* L.) as well as isolated seedlings of pedunculate oak (*Quercus robur* L.) in the understorey. However, beech seedlings are only found at locations with increased soil water content, whereas small oak seedlings are very rare. Both species did not occur at the study plots. At all plots, the soil type was classified as podzol (FAO classification) on Aeolian sand with mostly fine to medium sand (0.2–0.63 mm) of glacial origin as parent rock material with little gravel. The average annual air temperature at the weather station “Lindenberg” [~40 km distance to the field sites is 9.2°C with an average annual total precipitation of 576 mm (1981–2010, German Federal Meteorological Service (DWD)]. See also Table 1 for more details on stands and soil (Ah horizon) of the study sites. Sampling and field measurements were conducted in 2014, 1 year after nun moth population culmination, at four time points related to the developmental stages of the nun moth: (1) pre-defoliation stage in early May (spring), (2) main defoliation stage in late May, (3) post-defoliation stage in July (summer), and (4) later post-defoliation stage in early October (autumn). For N in throughfall, dry matter input, and soil leachates, samples were additionally taken at two winter dates: pre-defoliation in February and post-defoliation in November. The chosen sampling times during one vegetation period were based on the nun moth's development cycle: Adult nun moths fly from mid-July to the beginning of September and lay 70–300 eggs in bark alcoves with larvae hatching at the beginning of May and go through 5–7 larvae stages before pupation in July (Lipa and Glowacka, 1995). Newly hatched nun moth larvae prefer young, soft needles while older larvae also feed on old needles (Lipa and Glowacka, 1995). The feeding activity is very destructive because the needles upper half is cut off and then the remaining part is consumed (Lipa and Glowacka, 1995). A mass outbreak of the nun moth usually implies the economic end of the stand because often at least 50% of the trees die as a consequence of enormous needle loss (Eberswalde Forestry State Center of Excellence, 2013). Even after the insect mass outbreak, an increased vulnerability to storm events and drought stress remains, and under the current climate conditions in the northern German lowland, surviving trees usually require 5–6 years to regenerate completely (Eberswalde Forestry State Center of Excellence, 2013).

**Table 1:** Stand and soil details at the field sites.

	**Infested site**	**Control site**
**STAND**		
Tree height, average (m)	19	18
Tree age (years)	65	65
Stem density/ha	480	470
Diameter breast height (DBH, m)	23	23
**SOIL–Ah HORIZON**		
C content (%)	21.74	13.81
N content (%)	0.74	0.46
C/N ratio	29.42	30.25
pH (H_2_O)	3.4	3.3
pH (KCl)	2.4	2.6

*Average tree height (m), tree age (years), stem density per ha, and diameter at breast height (m) in the year of the study (forester, personal communication), C and N content (%), C/N ratio, and pH were determined in mixed soil samples of the Ah horizon in early May (n = 3). C and N were quantified in oven-dried, finely ground soil samples using an Elementar Vario EI analyser (Elementar Analysensysteme GmbH, Langenselbold, Germany). pH was measured on air-dried soil samples*.

### ^15^N uptake experiments

^15^N-labeling experiments were conducted in the field to quantify net N uptake capacity of infested and uninfested Scots pine trees. Per plots, 12 adult trees were randomly chosen. Intact fine roots (<2 mm diameter) still attached to the tree were carefully dug out, cleaned with water and incubated in 5 ml of an artificial soil solution according to the method described by Simon et al. (2010). The artificial soil solution contained 100 μM KNO_3_, 90 μM ${\text{CaCl}}_{2}^{*}$2 H_2_O, 70 μM MgCl_2_^*^ 6 H_2_O, 50 μM KCl, 24 μM ${\text{MnCl}}_{2}^{*}$ 4 H_2_O, 20 μM NaCl, 10 μM AlCl_3_, 7 μM ${\text{FeSO}}_{4}^{*}$ 7 H_2_O, 6 μM K_2_HPO_4_, 1 μM NH_4_Cl, 25 μM glutamine, and 25 μM arginine (pH 6.5), mimicking the conditions at a low N availability field site (Simon et al., 2013). Arginine and glutamine were chosen, because they represent the most abundant free amino acids in most plant parts (Griffin et al., 1991; Gessler et al., 1998). To quantify inorganic and organic net N uptake capacity, four different solutions were used for each individual tree, each containing all four N sources: ${\text{NH}}_{4}^{+}$, ${\text{NO}}_{3}^{-}$, as well as the amino acids glutamine and arginine. However, only one N form was labeled with ^15^N. Furthermore, a control solution without ^15^N-labeled compounds was used to determine the natural abundance of ^15^N in the roots. To avoid diurnal variation in N uptake (Gessler et al., 2002), incubation experiments were conducted for 2 h between 10 a.m. and 2 p.m. Following incubation, the submersed root parts plus an additional 10–15 mm were cut off, washed twice with 0.5 μM CaCl_2_, dried with cellulose paper, and the fresh weight was determined. After drying at 60°C for at least 48 h, the dry weight was determined.

### Sampling of fine root and needles

For the quantification of root and needle N metabolites, samples were taken from the same individuals used for ^15^N uptake experiments. Approximately 2–3 g of fine roots were sampled from three roots. In addition, 50–60 fresh previous-year needles were taken from different tree branches from the outer middle crown. All samples were immediately shock-frozen in liquid nitrogen and stored at −80°C on return from the field until further analyses.

### Sampling of insect feces, needle litter, throughfall, and soil solution

N input into the soil was measured as dry matter input (i.e., total N in insect feces and needle fragments), and in throughfall as well as soil solution [i.e., total N, nitrate-N, and dissolved organic N (DON), respectively] according to Le Mellec et al. (2011). At all plots, 10 randomly distributed throughfall samplers (diameter 20 cm) were set up. At each sampling date, samplers from each plot were pooled to five mixed samples. For collection of soil percolates, zero tension humus lysimeters were established underneath the humus layer at each plot according to Le Mellec et al. (2011). Sampling of throughfall and soil solution was conducted at biweekly intervals, during main defoliation at weekly intervals. Insect derived fragments (i.e., feces, leaf debris) and (green) litter fall were collected using nylon tree nets (mesh size 300 × 300 μm) with a net size between 15 and 17 m^2^ according to the canopy diameter (Le Mellec and Michalzik, 2008). Net sampling was conducted weekly.

### Quantification of ^15^N, ^13^C, and total N and C

To quantify ^15^N, ^13^C, as well as total N and C contents in the fine roots and total N content in needles, plant tissues were finely ground using a ball mill (Retsch TissueLyser, Haan, Germany). Aliquots of 0.8–2.5 mg were weighed into 4 × 6 mm tin capsules (IVA Analysentechnik, Meerbusch, Germany). Samples were analyzed with an elemental analyzer (NA2500, CE Instruments, Milan, Italy) coupled to an isotope ratio mass spectrometer (Delta Plus, Thermo Finnigan MAT GmbH, Bremen, Germany). As a working standard, glutamic acid was used, for δ^13^C calibrated against the primary standards USGS 40 (glutamic acid, δ^13^C_PDB_ = −26.39) and USGS 41 (glutamic acid, δ^13^C_PDB_ = 37.63) and for δ^15^N against USGS 25 (ammonium sulfate, δ^15^N_Air_ = −30.4) and USGS 41 (δ^15^N_Air_ = 47.600). To detect a potential instrument drift over time standards were included after every 12th sample. Net N uptake capacity was calculated according to Gessler et al. (1998): net N uptake capacity = ((^15^N_*l*_-^15^N_c_)^*^${\text{N}}_{\text{tot}}^{*}$dw^*^10^5^)/(MW^*^fw^*^t), where ^15^N_*l*_ and ^15^N_c_ represent the atom% of ^15^N in labeled (N_*l*_) and n control roots (i.e., natural abundance), respectively. N_tot_ is total N%, dw the dry weight, and fw the fresh weight of the root. MW is the molecular weight (^15^N g mol^−1^) and t stands for the incubation time. For each amino acid, the ^15^N/^13^C ratio of root fresh weight was compared to the total C/N ratio to determine whether amino acids were taken up as intact molecules (data not shown). Because net C uptake capacity based on ^13^C incorporation in root fresh weight differed from the ^15^N incorporation, it can be assumed that amino acids were either partially degraded in the artificial soil solution or on the root surface, or that amino acid-derived carbon was respired inside the roots (Simon et al., 2011).

### Quantification of total soluble protein-N, total amino acid-N, ammonium-N, nitrate-N, and structural N content in fine roots and needles

Before analyses of N metabolites, fine root and needle samples were ground to a homogeneous powder in liquid N_2_. Total soluble protein, amino acids, ammonium and nitrate concentrations were quantified according to Simon et al. (2010). For total soluble proteins content, ~0.05 g ground frozen plant material were extracted in 1.5 ml buffer containing 50 mM Tris-HCl (pH 8.0), 1 mM EDTA, 15% glycerol (v/v), 5 mM dithiothreitol, 0.1% Triton X-100 (v/v), 2 tablets of protease inhibitor cocktail (EDTA-free, Complete, Roche Diagnostics, Mannheim, Germany) and quantified using Bradford Reagent (Amresco Inc., Solon, Ohio, USA). The absorption was determined at 595 nm with a spectrophotometer (Ultrospec 3100 pro, Amersham Biosciences, Piscataway, USA). Bovine serum albumin (BSA) was used as standard. For total amino acids content, ~0.05 g finely ground plant tissue was extracted as previously described in 1 ml methanol-chloroform (3.5:1.5, v:v) and 0.2 ml Hepes buffer (20 mM Hepes, 10 mM NaF, 5 mM EGTA, pH 7.0) (Winter et al., 1992). The concentration of total amino acids was quantified using the method of Liu et al. (2005). Absorption was measured at 570 nm in a spectrophotometer (Ultrospec 3100 pro, Amersham Biosciences, Piscataway, USA). L-glutamine was used as standard. Ammonium and nitrate contents were quantified with the method reported by Simon et al. (2010): Approximately 0.04 g of plant tissue were soaked in 0.1 g washed polyvinylpyrrolidone (PVP, Sigma-Aldrich Inc., Steinheim, Germany) prepared in 1 ml distilled water to bind phenols. Ammonium and nitrate concentrations were determined using an ion chromatograph (DX 120, Dionex, Idstein, Germany) coupled to an autosampler (AS 3500, Thermo Separation Products, Piscataway, NJ, USA) equipped with the PeakNet software (version 4.3, Dionex). Anion mixtures of ${\text{NO}}_{3}^{-}$, ${\text{PO}}_{4}^{3-}$, ${\text{SO}}_{3}^{2-}$, and ${\text{SO}}_{4}^{2-}$ or cation mixtures of NH^+^, K^+^, Mg^2+^, and Ca^2+^, both in distilled water, were used as standards. Ammonium and nitrate contents of fine roots and needles were negligible (data not shown). Thus, structural N was calculated by substracting total soluble protein-N, and amino acid-N from total N.

### Quantification of N in insect feces, needle litter, as well as throughfall, and soil solution

N input into the soil was measured in dry matter (i.e., total N in insect feces and needle litter), throughfall as well as soil solution (i.e., total N, nitrate-N, and dissolved organic N each). For determination of total N in dry matter, insect feces were separated from needle litter and both dried at 45°C. Fresh and dry weight was determined. Total N content was quantified in aliquots of the dry material (3 technical replicates per net) by thermal oxidation using a Leco CHN 1000 analyser (LECO Enterprise, Mönchengladbach, Germany). Aliquots of throughfall and soil solutions were filtered with 0.45 μm cellulose-acetate membrane filters (Sartorius, Göttingen, Germany) and analyzed for dissolved N (DN) via thermal oxidation (Dimatoc 100, Dimatex, Essen Germany) and for nitrate by ion chromatography (761 Compact IC, Methrom, Filderstadt, Germany) according to Le Mellec et al. (2011). DON was calculated as difference between DN and nitrate-N. Total N was quantified by thermal oxidation (Dimatoc 100, Dimatec, Essen, Germany) in solutions of 0.45 and 500 μm particle size.

### Statistical analyses

Prior to comparisons between treatments, all data were tested for normal distribution and homogeneity of variances. Statistical analyses for inorganic and organic net N uptake capacity as well as N metabolites in roots and needles were performed using R package version 1.3.1 (R Development Core Team, 2008). Differences between infested and control sites were detected using Kruskal–Wallis test on ranks. For multiple pairwise comparison within one treatment at different sampling dates between the different N sources as well as N metabolites and infested vs. control plots at each sampling date, *post-hoc* Dunn's test was performed using the Bonferroni correction for *p*-value adjustment (Dinno, 2015). For statistical analyses of throughfall, dry matter, and soil leachates, SPSS (SPSS Statistics for Windows, version 22.0., IBM, Armonk, USA) was used. Comparisons between infested and control sites, as well as sampling dates were tested using ANOVA.

## Results

### Insect mass outbreak affects N fluxes in dry matter input, throughfall, and soil leachates

In general, mean and accumulated N input into the soil (i.e., total N in insect feces, litter, and throughfall, as well as DON in throughfall) was significantly higher at the infested compared to the control plots at all sampling times (Table 2). Similarly, N output (i.e., total N, nitrate-N, and DON in soil leachates) was significantly higher with insect infestation regardless of sampling time. Only DON in soil solution (winter I and II) and nitrate-N fluxes in throughfall (entire year) did not differ significantly in response to insect infestation. With regard to N allocation in throughfall, DON fluxes were generally higher than nitrate-N (*p* ≤ 0.050), whereas soil leachates showed a reversed pattern with higher nitrate-N compared to DON (*p* ≤ 0.050). Comparison with N fluxes across the sampling times showed no significant differences for the control plots. In contrast, for the infested plots, total N, nitrate-N, and DON in throughfall as well as DON in soil leachates increased significantly from the previous winter sampling to the main defoliation event (*p* ≤ 0.050). For total N and nitrate-N in soil leachates, a significant increase was delayed (i.e., from previous winter to autumn sampling; *p* ≤ 0.050).

**Table 2:** Mean N fluxes (kg/ha) per month and cumulative N fluxes (kg/ha) per 6 months.

**Mean N fluxes (kg ha^−1^)**								
	**totN feces**	**totN litter**	**totN throughfall**	**NO throughfall_3_-N**	**DON throughfall**	**totN soil solution**	**NO soil solution_3_-N**	**DON soil solution**
**INFESTED**								
Winter I	n.a.	n.a.	14.3 ± 6.5^*^A	3.1 ± 0.8A	6.5 ± 2.9^*^A	7.5 ± 4.3^*^A	6.3 ± 2.1^*^A	0.7 ± 0.1A
Spring	n.a.	n.a.	16.8 ± 7.9^*^AB	3.7 ± 1.3AB	6.8 ± 3.2^*^AB	9.7 ± 4.9^*^AB	8.1 ± 3.1^*^AB	0.8 ± 0.1^*^AB
Main defoliation	11.3 ± 2.9^**^A	5.9 ± 1.9^**^A	17.9 ± 8.1^*^B	4.7 ± 1.6B	11.3 ± 2.9^*^B	10.7 ± 5.1^*^AB	9.5 ± 3.4^*^AB	1.1 ± 0.2^*^B
Summer	8.9 ± 3.6^*^A	6.7 ± 2.4^**^A	15.4 ± 8.3^*^AB	3.9 ± 1.3AB	8.7 ± 3.6^*^AB	10.8 ± 4.8^*^AB	9.6 ± 3.4^*^AB	1.0 ± 0.1^*^AB
Autumn	5.7 ± 3.4^**^A	6.3 ± 2.1^**^A	14.9 ± 7.1^*^AB	4.4 ± 1.7AB	7.8 ± 3.4^*^AB	10.9 ± 4.9^*^B	10.0 ± 3.6^**^B	0.9 ± 0.3^*^AB
Winter II	n.a.	6.5 ± 1.9^**^A	12.3 ± 7.1^*^AB	3.3 ± 0.8AB	6.9 ± 2.3^*^AB	9.7 ± 3.9^*^AB	8.3 ± 2.9^**^AB	0.7 ± 0.2AB
Mean	8.6 ± 3.3^**^	6.4 ± 2.1^**^	15.3 ± 7.5^*^	3.9 ± 1.3	8.0 ± 3.1^*^	9.9 ± 4.7^*^	8.6 ± 3.1^*^	0.9 ± 0.2^*^
Sum	25.9 ± 2.8^**^	25.4 ± 0.3^**^	91.6 ± 2.0^**^	23.1 ± 0.6	48.0 ± 1.8^**^	59.3 ± 1.3^*^	51.8 ± 1.4^*^	5.2 ± 0.2^*^
**CONTROL (NON-INFESTED)**								
Winter I	n.a.	n.a.	9.9 ± 3.9^*^A	4.3 ± 1.8A	5.3 ± 1.9^*^A	6.7 ± 3.4^*^A	5.9 ± 1.9^*^A	0.6 ± 0.1A
Spring	n.a.	n.a.	11.1 ± 3.3^*^A	4.6 ± 1.9A	5.4 ± 1.3^*^A	6.9 ± 3.2^*^A	5.6 ± 1.6^*^A	0.5 ± 0.1^*^A
Main defoliation	3.3 ± 0.5^**^A	1.4 ± 0.6^**^A	11.6 ± 3.6^*^A	4.5 ± 2.0A	5.6 ± 1.2^*^A	7.1 ± 3.2^*^A	5.7 ± 1.8^*^A	0.4 ± 0.1^*^A
Summer	3.6 ± 0.7^**^A	1.4 ± 0.4^**^A	10.6 ± 3.4^*^A	4.6 ± 1.3*A*	5.3 ± 1.3^*^A	6.3 ± 3.5^*^A	5.6 ± 1.7^*^A	0.1 ± 0.1^*^A
Autumn	3.7 ± 0.7^**^A	1.5 ± 0.5^**^A	9.8 ± 3.4^*^A	4.1 ± 2.3*A*	5.1 ± 0.9^*^A	6.9 ± 3.0^*^A	4.9 ± 1.9^**^A	0.1 ± 0.1^*^A
Winter II	n.a.	1.4 ± 0.2^**^A	10.1 ± 3.9^*^A	4.7 ± 2.3A	4.9 ± 2.0^*^A	6.9 ± 3.3^*^A	4.9 ± 1.2^**^A	0.1 ± 0.1A
Mean	3.5 ± 0.6^**^	1.4 ± 0.4^**^	10.5 ± 3.6^*^	4.5 ± 1.9	5.3 ± 1.4^*^	6.8 ± 3.3^*^	5.4 ± 1.6^*^	0.5 ± 0.1^*^
Sum	10.6 ± 0.2^**^	5.7 ± 0.1^**^	73.6 ± 0.7^**^	26.8 ± 0.2	31.6 ± 0.2^**^	40.8 ± 0.3^*^	32.6 ± 0.4^*^	3.0 ± 0.3^*^

totN, total N; DON, dissolved organic N; Mean, average N fluxes over a 6 months period; sum, sum of N fluxes over a 6 months period. Asterisks indicate level of significance between control and infested plots (

* *p < 0.050*,

** *p < 0.010). Different capital letters indicate significant differences between sampling times within one treatment (p ≤ 0.050). n.a. = not available*.

### Insect mass outbreak reduced organic and inorganic net N uptake capacity of fine roots in scots pine

**Figure 1:**
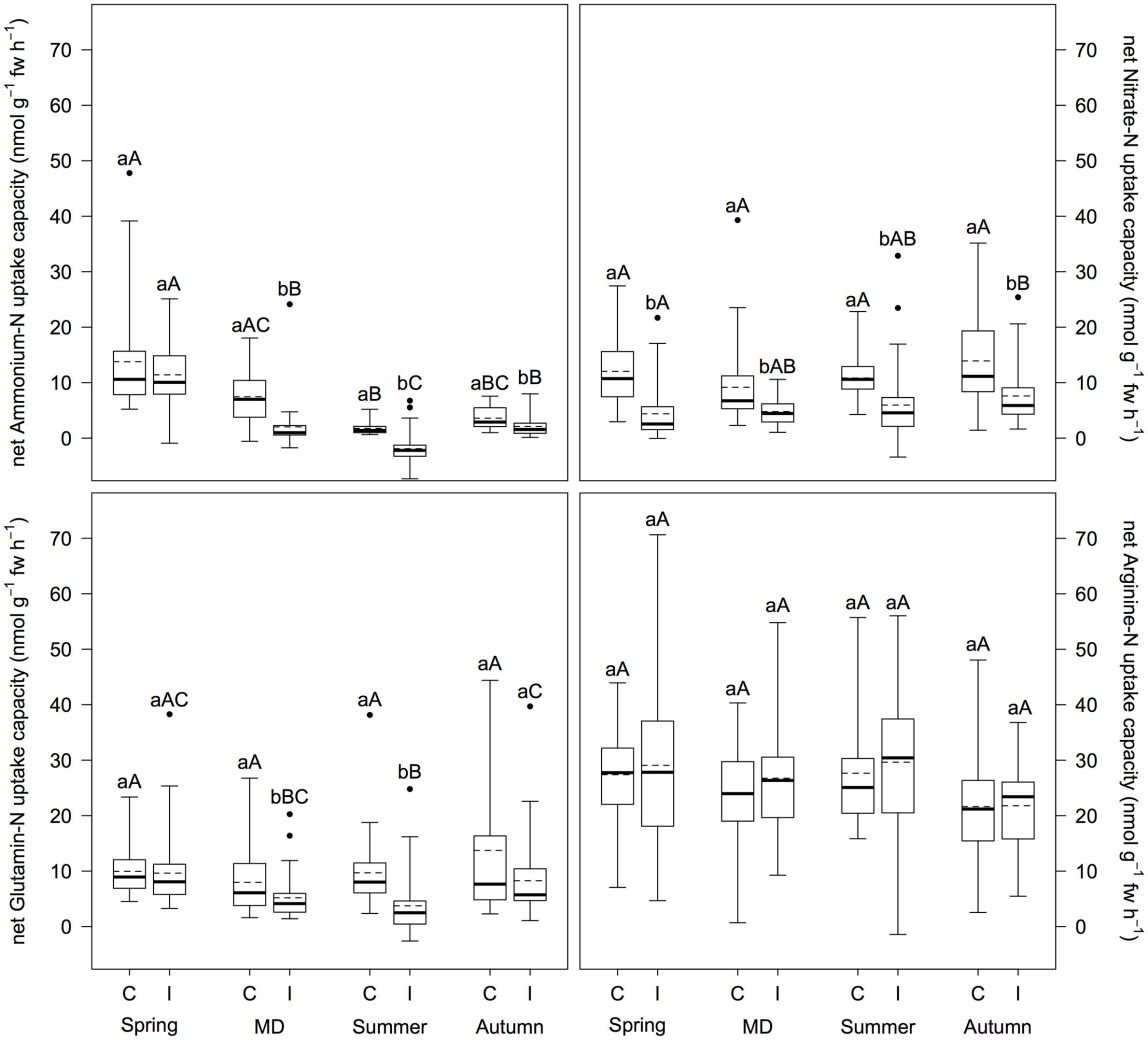
Inorganic and organic net N uptake capacity (nmol g^−1^ fw h^−1^) at infested and non-infested pine forests over the vegetation period. I, infested; C, control; MD, main defoliation. Box plots show means (dotted lines) and medians (straight lines) (*n* = 12 for each plot). Whisker extension equals 3x interquantile range distance. Different small letters indicate significant differences between infested and control plots within one sampling time (*p* ≤ 0.050). Different capital letters indicate significant differences between the sampling times within one treatment (*p* ≤ 0.050).

Mass outbreaks of defoliating nun moth affected net N uptake capacity of Scots pine differently depending on N source (Figure 1). Net uptake capacity of ammonium-N, nitrate-N, and glutamine-N (i.e., only for main defoliation and summer) was significantly reduced in trees of the infested stands compared to the control stands (*p* < 0.001), whereas arginine-N net uptake capacity was not influenced at all. Mean reduction of net ammonium-N, nitrate-N, and glutamine-N uptake capacity was 29.6, 65.3, and 65.4%, respectively. Furthermore, net N uptake capacity changed over the vegetation period depending on N source. Net ammonium-N uptake capacity regardless of insect infestation and net glutamine-N uptake capacity at the infested plots were highest in spring (*p* ≤ 0.007), however, decreased during main defoliation and summer with another increase in autumn (*p* < 0.020). Net arginine-N uptake capacity decreased regardless of insect infestation from summer to autumn (*p* ≤ 0.042). For net nitrate-N uptake capacity no differences over the vegetation period were found. With regard to preference for certain N sources, we found differences between treatments: At the control plots, arginine-N was preferred over nitrate-N, glutamine-N, and ammonium-N (*p* ≤ 0.010); at the infested plots arginine-N was preferred over glutamine-N and nitrate-N over ammonium-N (*p* ≤ 0.001).

### Insect mass outbreak enhanced total N, soluble protein-N, amino acid-N, and structural N in fine roots and needles of scots pine

**Figure 2:**
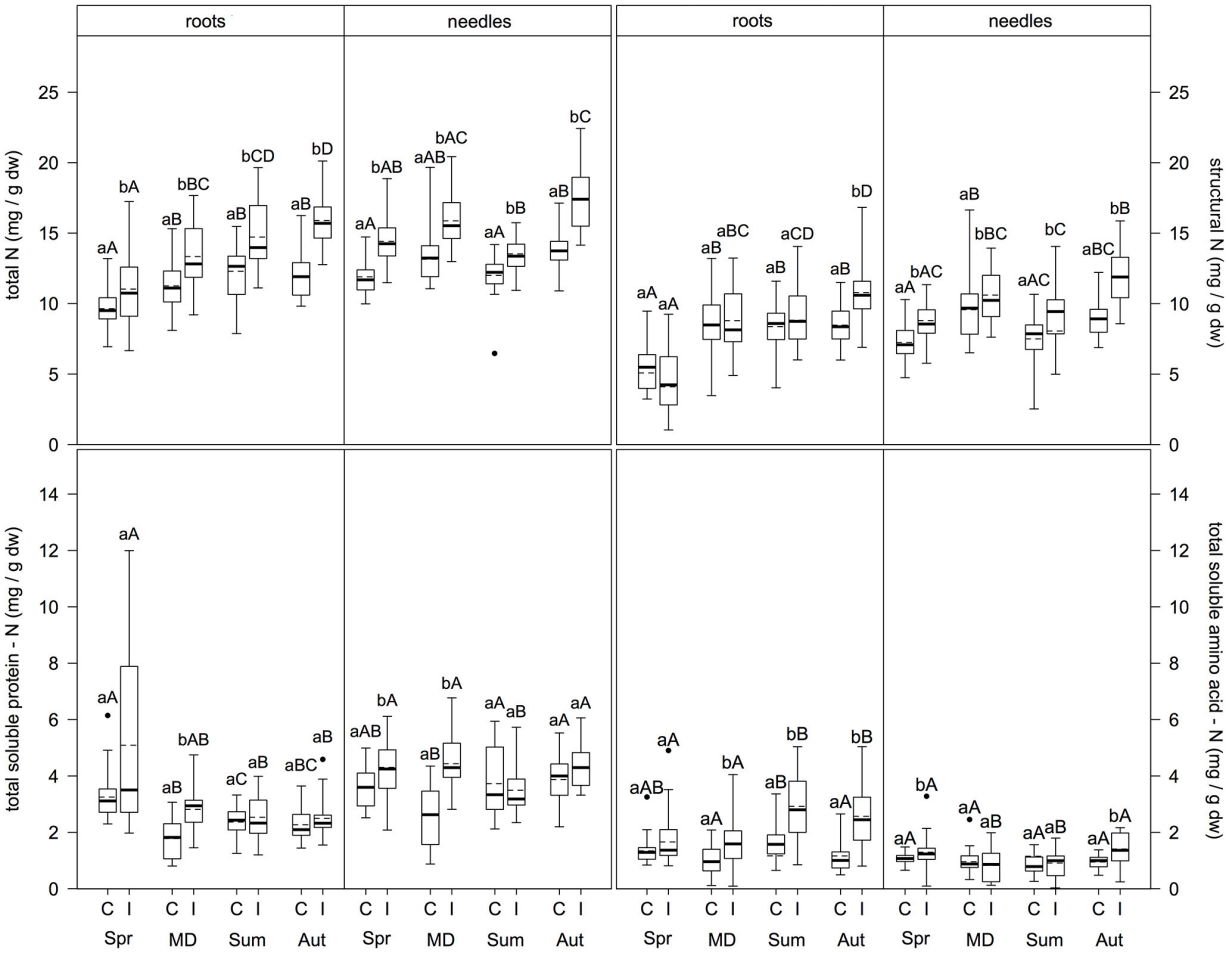
Total N, structural N, soluble protein-N, and soluble amino acid-N content in fine roots and needles (mg/g dw) at infested and non-infested pine forests over the vegetation period. I, infested; C, control; MD, main defoliation. Box plots show means (dotted lines) and medians (straight lines) (*n* = 12 for roots, *n* = 8 for needles for each plot). Whisker extension equals 3x interquantile range distance. Different small letters indicate significant differences between infested and control plots within one sampling time (*p* ≤ 0.050). Different capital letters indicate significant differences between the sampling times within one treatment (*p* ≤ 0.050).

In the fine roots (Figure 2), N metabolite concentrations were significantly higher at infested compared to control plots for total N (regardless of sampling time; *p* ≤ 0.009), structural N (only in autumn; *p* < 0.001), total soluble protein-N (TSP-N, only at the main defoliation event; *p* < 0.001), and total soluble amino acid-N (TAA-N, at all sampling times except spring; *p* ≤ 0.001). Needles showed higher concentrations at infested compared to control plots for total N and structural N (both regardless of sampling time; (*p* ≤ 0.001 and *p* ≤ 0.031, respectively), TSP-N (only in spring and at the main defoliation event (*p* ≤ 0.003), and TAA-N (only in spring and autumn; (*p* ≤ 0.016).

Comparing sampling times within each parameter, levels of N metabolites differed between sampling dates (Figure 2). In fine roots of control plots, both total N and structural N concentrations did not differ significantly between the main defoliation event, summer, and autumn, but were significantly higher at these time points compared to spring (*p* ≤ 0.046 and *p* ≤ 0.001, respectively). TSP-N concentration was highest in spring > summer/autumn > main defoliation (*p* ≤ 0.001). Similarly, TAA-N concentrations were highest in summer compared to the main defoliation event and autumn (*p* ≤ 0.008) with no differences between spring and the other sampling times. For the infested plots, fine roots showed different patterns: Total N concentration was higher in autumn > main defoliation event > spring (*p* ≤ 0.001), with concentrations in summer only higher compared to spring (*p* ≤ 0.001) and no differences between the other sampling dates. Structural N levels were highest in spring > frass > autumn (*p* ≤ 0.001) and lower in summer compared to spring (*p* ≤ 0.001). TSP-N concentrations were highest in spring > summer/autumn (*p* ≤ 0.001), but no significant differences between any of the other time points. TAA-N concentrations were significantly higher in summer/autumn compared to the main defoliation event and spring (*p* ≤ 0.007). Levels of N metabolites showed different patterns over the vegetation period in the leaves. At the control plots, total N concentrations were higher in autumn compared to summer and spring (*p* ≤ 0.016), with no differences at the main defoliation event compared to the other sampling times. Structural N concentration was highest, whereas TSP-N concentration was lowest at the main defoliation event (*p* ≤ 0.016). TAA-N concentration did not change at all between sampling times. At the infested plots, needles showed different patterns with regard to their N metabolites: Total N content in the needles was lower in summer compared to the main defoliation event and autumn and lower in spring compared to autumn (*p* < 0.008), but no other significant differences were found. Structural N concentration was higher with frass and in autumn compared to spring (*p* ≤ 0.019), and lower in summer compared to autumn (*p* = 0.005). TSP-N was lowest in summer (*p* ≤ 0.040) with no differences between the other sampling times. TAA-N levels were higher in spring and autumn compared to the main defoliation event and summer (*p* ≤ 0.043).

Comparing composition of N pools in fine roots and needles, total N mainly consisted of structural N, with 59 ± 10.8% in roots of infested and 65 ± 8.4% control trees, and 64 ± 4.2% in needles of infested and 61 ± 10.4% of control trees. The second largest contribution was TSP-N: 24 ± 12.4% in roots of infested and 21 ± 7.9% in control trees, and 26 ± 2.9% in needles of infested and 30 ± 9.1% of control trees. The lowest contribution was from TAA-N with 15 ± 3.1% in roots of infested and 11 ± 2.4% of control trees, and 9 ± 4.3% in needles of infested and 8 ± 1.5% of control trees. Comparing N metabolites in fine roots and needles over the vegetation period, total N and structural N contents in needles were higher in summer and autumn compared to spring and main defoliation in both, infested and control trees (*p* ≤ 0.001 and *p* ≤ 0.004). TSP-N in the needles was higher compared to the roots at the main defoliation event, summer and autumn sampling times (*p* ≤ 0.001). In contrast, TAA-N levels in the roots of infested trees were higher than in needles (*p* ≤ 0.001), except for spring. However, in spring and summer root TAA-N levels of the control trees were higher than in needles (*p* ≤ 0.004).

## Discussion

### Consequences of insect mass outbreaks on soil N fluxes

#### Insect mass outbreaks in scots pine stands lead to enhanced N input and nitrate leaching

Insect mass outbreaks can affect N cycling directly via changes in quantity and quality—specifically that of organic input of feces and dead insect biomass as well as changes in throughfall composition (Stadler et al., 2005; Müller et al., 2006; Le Mellec and Michalzik, 2008)—and indirectly via changes in rhizodeposition, modified nutrient uptake rates by trees and altered root-soil microbe-interactions (Zimmer and Topp, 2002; Throop and Lerdau, 2004). In the studied Scots pine forests, the insect mass outbreak altered soil N cycling resulting in enhanced N input (i.e., total N, nitrate-N, DON) via feces and litter as well as N output (i.e., total N, nitrate-N, DON) compared to the control plots. In other studies, higher total N fluxes in throughfall were mainly due to increased fluxes of DON originating from leaching of damaged leaves and washouts of branches and leaves with phyllosphere microorganisms (Hunter, 2001; Le Mellec and Michalzik, 2008). For example, growth of epiphytic heterotrophic microorganisms was increased on infested needles and leaves of spruce and oak trees which was triggered by sugars and carbon rich excretions from insects. In turn, microbial growth in the phyllosphere changed the N composition of the throughfall (Guggenberger and Zech, 1994; Stadler and Michalzik, 2000). The increase in total N of litter and feces at infested plots compared to the controls was due to quantitative higher organic entries. The additional N input via feces into the system might have further implications. Feces consist mainly of labile C as well as extractable N in form of proteins which can stimulate soil microbial activity resulting in increased CO_2_ emissions from the soil (Lovett and Ruesink, 1995; Zimmer and Topp, 2002; Frost and Hunter, 2004). Because the physical and chemical structure of feces is easily soluble compared to needle litter (Jung and Lunderstädt, 2000), feces are likely to mediate faster N turnover in the soil. The increased N input with insect mass herbivory in the present study also explains the increased N output as total N, nitrate-N, and DON at the infested forest site, because additional organic input leads to an enhanced release of nutrients via the soil solution (Stadler et al., 2001; Chapman et al., 2003; Le Mellec et al., 2009, 2011). Enhanced N output during insect mass outbreaks was previously observed for other forests and pest insects (e.g., Swank et al., 1981; Näsholm, 1994; Houle et al., 2009; Pitman et al., 2010). For example, in a boreal forest, the inorganic N output via soil the solution was 30 times higher during a mass outbreak of the spruce budworm compared to an undisturbed forest site (Houle et al., 2009).

#### Consequences of an insect mass outbreak on soil N fluxes are linked to the life cycle of the feeding insects

Variation in N fluxes over the vegetation period were observed only at the infested, but not the control plots suggesting that changes at the infested plots were caused by the nun moth and the related biotic stress. The observed N input fluxes increased from winter to spring and peaked at the main defoliation event, during which nun moth larvae are most active, and declined again until autumn, while the nun moth pupated, metamorphosed, and mated. The higher total N and DON fluxes in throughfall at the beginning of the previous winter compared to the winter following the main frass activity at the end of the measuring period are likely a response to the previous year's infestation. Similar patterns of increased N input via litter and throughfall over the vegetation period as a response to insect mass outbreaks have been reported in other studies (Stadler et al., 2001; Le Mellec et al., 2009; Pitman et al., 2010), suggesting that this response to massive herbivory is strongly linked to the variation in the life cycle of the feeding insects over the vegetation period.

N fluxes in the soil solution showed a delayed response to insect mass outbreaks with fluxes of total N, nitrogen-N, and DON peaking in autumn. This can be explained by several processes: (1) The reaction-time of a soil system is mainly regulated by rainfall events and soil buffer capacity which depends on the soil N status prior to the outbreak event (Pitman et al., 2010; Griffin et al., 2011). Soils with low N availability, such as the present study site, often show a lower or delayed reaction with regard to N input in the soil solution compared to N-saturated soils due to higher microbial immobilization that incorporates N into stable organic matter (Frost and Hunter, 2004, 2007). (2) Furthermore, the export of N via nitrate and DON depends on soil water availability. At our research site, water supply is limited during late spring and summer due to low precipitation and high temperatures (Grüning, personal observation; Gerstengarbe et al., 2003). Increasing precipitation and decreasing transpiration in autumn and winter contribute to the peak of N fluxes in the soil solution in autumn. These results show the significance of sampling time for soil N cycling. Overall, additional N input in response to insect mass herbivory exceeded N losses via leaching. Thus, insect mass outbreaks have the potential to cause long-term effects on soil N cycling by significantly increasing the total N load in soils (Vestgarden, 2001), and thus providing an additional N source for understorey vegetation and/or tree regeneration in the years following the insect mass outbreak (Griffin et al., 2011; Kaňa et al., 2013).

### Consequences of insect mass outbreaks on tree N nutrition

#### Reduction of N acquisition of inorganic N and glutamine-N by the roots

Tree N uptake strongly depends on soil N availability (Stoelken et al., 2010; Simon et al., 2013; Dong et al., 2016). N supply in soils of infested forests is often increased (Belovsky and Slade, 2000; Chapman et al., 2003; Le Mellec and Michalzik, 2008) as also found in the present study (see above). Still, inorganic N and glutamine-N net uptake capacity of Scots pine trees were strongly reduced (30–65% reduction depending on N source) under massive herbivory by the nun moth, especially at the main defoliation event. A reduced inorganic N uptake capacity was found also for hybrid poplar (*Populus x canadensis* cv. Eugeneii) defoliated by gypsy moths (Kosola et al., 2001). In contrast, for defoliated oak seedlings (Lovett and Tobiessen, 1993) and hemlock saplings (Rubino et al., 2015) a difference in inorganic net N uptake was not detected. Three aspects differed in the latter two studies compared to the present study: (1) Rubino et al. (2015) applied ^15^N directly to the soil, (2) both studies used only inorganic N sources, and (3) both studies investigated seedlings instead of adult trees. However, N acquisition strongly varies with tree age (Simon et al., 2011). For example, woody seedlings take up inorganic and organic N preferably in spring, whereas adult beech trees show highest N uptake in autumn (Simon et al., 2011). Furthermore, loss of needle biomass, and thus photosynthetic tissues, due to massive insect herbivory raises the relative costs of root N uptake (Jacquet et al., 2014; Fang et al., 2016). For the utilization of amino acid-N, less energy is on average required compared to inorganic N sources such as nitrate and ammonium (Zerihun et al., 1998; Gruffman et al., 2013). The preference for arginine-N in the present study supports the view that organic N sources might be preferred over inorganic N sources at limited energy generation by photosynthesis. However, under these conditions internal reallocation of N might even be a better strategy for survival than organic N uptake. Therefore, it is not surprising that infested Scots pine trees did not use the additional N available in the soil upon infestation in the present study.

Previous assumptions that Scots pine has a reduced affinity toward glutamine and prefers ammonium as N source (Persson and Näsholm, 2003; Simon et al., 2013) were not confirmed by the present results. The shift of N uptake toward glutamine-N by infested trees was mainly caused by a reduction in nitrate rather than an increase in glutamine-N uptake capacity with infestation. The observed preference of Scots pine for organic N sources has been described previously by Persson et al. (2006) and Simon et al. (2013). It is also relevant for other species, such as European beech (Dannenmann et al., 2009; Stoelken et al., 2010; Simon et al., 2011; Li et al., 2015), oak, hemlock (Gallet-Budynek et al., 2009), willow, and black spruce (Kielland et al., 2006).

#### Consequences of an insect mass outbreak on organic and inorganic N acquisition depend on sampling time over the vegetation period

The uptake of ammonium-N and glutamine-N by infested Scots pine is influenced by sampling time during the vegetation period and thereby strongly linked to the life cycle of the nun moth. N uptake capacity was reduced during the insect mass outbreak. Changes of inorganic and organic N uptake at different times during the vegetation period have been investigated in previous studies (Simon et al., 2011; Dong et al., 2016). However, in the present study the observed changes were only found for infested trees, but not for control trees, suggesting a link to the increasing population size of the nun moth with a population peak at the main defoliation event. Rising spring and summer temperatures and increased evapotranspiration might exacerbate the decline of external N acquisition, thereby adding stress to the already physiologically impaired trees (Heinzdorf, 2013). N uptake is strongly related to water as well as N availability (Gessler et al., 1998; Stoelken et al., 2010; Li et al., 2015; Dong et al., 2016). Furthermore, N acquisition is species-specific and depends on current tree N nutrition (Näsholm et al., 2009; Gruffman et al., 2014). The high ammonium net uptake capacity found in the present study in spring—when nun moth activity is still relatively low—suggests that insect mass herbivory already interacts with tree internal regulation of N acquisition at early insect infestation. The present data also suggest that infested trees might face difficulties replenishing their N reserves from external sources after winter by N uptake in case of an insect mass outbreak.

#### Enrichment of total N in roots and remaining needles of infested trees as a compensatory response to insect defoliation

In response to insect herbivory and defoliation, Scots pine trees accumulated N in fine roots and remaining needles. This increase in total N concentration is not a result of enhanced N acquisition from the soil (see above), but rather due to an increase in levels of total soluble protein-N, amino acid-N, and structural N that is meditated by tree internal sources. Second-year needles are the major storage pool for N in Scots pine trees (Millard et al., 2001). N stored in these needles might serve as buffer against short-term fluctuations in N supply (Millard and Grelet, 2010), for example when N acquisition by the roots is reduced during an insect mass outbreak. N in needles covers up to 60% of the tree's N demand for the next vegetation period, thus playing a key role in tree regeneration after partial defoliation (Millard and Grelet, 2010; Polacco and Todd, 2011). For example, a 50% defoliation of mountain beech (*Betula pubescens* ssp. *tortuosa* Ehrh.) by autumnal moth (*Epirrita autumnata* Borkhausen) lead to a N accumulation of the remaining leaves, most likely in form of Rubisco (Ribulose-1,5-bisphosphate carboxylase; Hoogesteger and Karlsson, 1992; Palacio et al., 2012), thereby stimulating the rate of photosynthesis of the remaining leaves as compensatory reaction (Lovelock et al., 1999). In general terms, the increase in N pools in needles and fine roots might serve different purposes: (1) Amino acid levels might increase in direct response to N fertilization in different plant parts of adult Scots pine (bark, wood, foliage: Nordin et al., 2001; needles: Näsholm and Ericsson, 1990; roots: Ahlström et al., 1988). (2) As a consequence of defoliation, tree water balance is disturbed (Bréda et al., 2006), because of an imbalance between root water uptake and leaf transpiration (Aroca et al., 2012). Accumulation of amino acids serving as osmoprotectants (Griffin et al., 1991) enables the tree to maintain water uptake and, consequently N supply, even when water-stressed (Fotelli et al., 2002; Rennenberg et al., 2006). (3) The N stored in the remaining needles in the canopy is also invested in the production of defense compounds, such as phenolics and lignin (Millard and Grelet, 2010; Fang et al., 2016). On the other hand, higher nitrogen contents can benefit herbivores by improving the nutritional quality of the host plant. However, plant defense compounds also depend on the trees N content, which in turn has negative impacts on the insect's growth and survival (Kytö et al., 1996). Furthermore, roots produce organic exudates (e.g., amino acids) against secondary infestations with pathogenic root feeding bacteria, fungi, and insects (Bezemer and van Dam, 2005; Oliva et al., 2016). Metabolites involved in aboveground defense are also synthesized in the roots and then transported aboveground (Van der Putten et al., 2001). In the study, the increase in structural N, i.e., lignin, in the fine roots that can also be considered a means of defense was seen only in autumn indicating a time-delayed response to defoliation. Similar to the present study, increased concentrations of total N, TAA-N, and TSP-N in needles were found in studies investigating eastern hemlock infested with hemlock wooly adelgid (e.g., Stadler et al., 2005; Gómez et al., 2012; Rubino et al., 2015; Soltis et al., 2015). With 72% we measured an even stronger increased TSP-N content in infested Scots pine needles during main defoliation compared to control trees. In contrast, TAA-N concentrations in needles were significantly higher only in spring and autumn, which could be the result of protein breakdown of infested needles during the main defoliation period (Krasensky and Jonak, 2012). The amino compounds produced could be allocated to non-infested needles thereby contributing to its enhanced N content. Apparently, defoliation by the nun moth has consequences at the whole-tree level and mediates responses not only in the most affected plant tissues (i.e., needles). When N acquisition by the roots is reduced as a response to insect mass herbivory, although soil N availability is high, internal reallocation of N seems to be a means to counteract the biotic stress.

Besides internal reallocation of N, tree carbon (C) resources and/or internal allocation might change in course of defoliation as net photosynthesis decreases when major parts of a tree's green tissue are lost (l-M-Arnold et al., 2016). Together with an accumulation of N in response to defoliation (as observed in the present study), C storage compounds might increase as well, as has been found for various tree species, such as black pine (*Pinus nigra* J.F.Arn., Palacio et al., 2012), eastern hemlock (*T. canadensis* L., Soltis et al., 2015), balsam fir (*Abies balsamea* L., Deslauriers et al., 2015), and red oak (*Q. rubra* L., Frost and Hunter, 2008). This increase in C might support re-growth after defoliation (Palacio et al., 2012) or serve as defense by thickening cell walls via accumulation of C-rich cellulose and lignin (Soltis et al., 2015). As a result C/N ratio would be more or less constant, and indeed this was found in a study by Le Mellec et al. (2009) for a Scots pine forest comparable to our study site, both belonging to the same region of periodically reoccurring insect mass outbreaks. Contrasting this, lower needle C/N ratios were observed in three pine species (*Pinus pinaster* A., *P. nigra* J.F.Arn., *P. sylvestris* L.) defoliated by the pine processionary moth (*Thaumetopoea pityocampa* Den.Schiff.) indicating an overbalance of N accumulation in comparison to C accumulation (Hódar et al., 2015).

Overall, nun moth defoliation has been shown to reduce annual tree growth of Scots pine, which can be explained by inhibited water and nutrient supply and reduced photosynthetically active tissue (Beker, 1996). With >90% needle loss in one vegetation period, a threshold is reached for Scots pine leading to significantly decreased tree growth and increased mortality rates (Cedervind and Langstrom, 2003), which might become even more severe with multiple consecutive years of severe defoliation (Van Asch and Visser, 2007).

## Conclusions

The studied insect mass outbreak had significant impact on forest soil N cycling as well as N nutrition of Scots pines. Both, N input and output of the humus layer in the forest soil were strongly related to the biomass loss in response to the massive insect herbivory. To compensate for the aboveground losses of biomass, trees can either increase root N acquisition for the stimulation of growth processes (Lovett and Tobiessen, 1993) or can reallocate N from internal sources. The present study suggests reallocation of N from internal sources, because inorganic and organic N acquisition of tree roots was reduced in spite of increased soil N availability, whereas total N, structural N, soluble amino acid-N, and soluble protein-N levels were increased in fine roots and the remaining needles. However, N in- and output fluxes in the soil and within trees vary depending on environmental factors, such as climate, soil type, insect population dynamics, and outbreak intensity (Jung, 2008; Gallet-Budynek et al., 2009; Keville et al., 2013). Our study investigated the consequences of an insect mass outbreak only for the duration of one vegetation period, but not potential recovery of the surviving trees. Thus, further studies are required to gain further insights into these complex processes.

## Author contributions

JS and Al conceived and designed the study. MG and JS conducted the ^15^N uptake experiments and analysis of N uptake capacity and N metabolites. Al sampled and analyzed N in throughfall, dry matter, and soil leachates. Data evaluation and manuscript writing was performed by MG, Al, and JS. HR contributed the IRMS analyses and ideas to the study and the manuscript. All authors agreed on the final version of the manuscript.

### Conflict of interest statement

The authors declare that the research was conducted in the absence of any commercial or financial relationships that could be construed as a potential conflict of interest. The reviewer SN and handling Editor declared their shared affiliation, and the handling Editor states that the process met the standards of a fair and objective review.
